# Prognostic microRNAs in high-grade glioma reveal a link to oligodendrocyte precursor differentiation

**DOI:** 10.18632/oncoscience.112

**Published:** 2014-12-26

**Authors:** Josie Hayes, Helene Thygesen, Alastair Droop, Thomas A. Hughes, David Westhead, Sean E. Lawler, Heiko Wurdak, Susan C. Short

**Affiliations:** ^1^ Leeds Institute of Cancer and Pathology, University of Leeds, St James's University Hospital, Leeds, UK; ^2^ Leeds Institute of Biomedical and Clinical Sciences, University of Leeds, St James's University Hospital, Leeds, UK; ^3^ Institute of Molecular and Cellular Biology, Faculty of Biological Sciences and Institute of Membrane and Systems Biology, Faculty of Biological Sciences, University of Leeds, Leeds, UK; ^4^ Department of Neurosurgery, Brigham and Women's Hospital, Harvard Medical School, Boston, MA, USA

**Keywords:** glioma, oligodendrocyte, glioblastoma, astrocytoma, microRNA, prognosis

## Abstract

MicroRNA expression can be exploited to define tumor prognosis and stratification for precision medicine. It remains unclear whether prognostic microRNA signatures are exclusively tumor grade and/or molecular subtype-specific, or whether common signatures of aggressive clinical behavior can be identified. Here, we defined microRNAs that are associated with good and poor prognosis in grade III and IV gliomas using data from The Cancer Genome Atlas. Pathway analysis of microRNA targets that are differentially expressed in good and poor prognosis glioma identified a link to oligodendrocyte development. Notably, a microRNA expression profile that is characteristic of a specific oligodendrocyte precursor cell type (OP1) correlates with microRNA expression from 597 of these tumors and is consistently associated with poor patient outcome in grade III and IV gliomas. Our study reveals grade-independent and subtype-independent prognostic molecular signatures in high-grade glioma and provides a framework for investigating the mechanisms of brain tumor aggressiveness.

## INTRODUCTION

Malignant gliomas comprise the grade III and IV gliomas as defined by the World Health Organization [1]. Glioblastoma multiforme (GBM, WHO Grade IV) has a median survival of 12-15 months and can arise *de novo*, or following progression from grade III disease/anaplastic astrocytoma (GIIIA), which has five-year survival rates of 24% [2,3]. Genomic biomarkers of malignant glioma include isocitrate dehydrogenase 1/2 (*IDH1/2)* mutations, O-6-methylguanine-DNA methyltransferase *(MGMT)* promoter methylation status and 1p19q co-deletion, and these markers provide information on prognosis and response to treatment [4-6]. It has also been shown recently that hypermethylation at a large number of loci, known as the glioma CpG island methylator phenotype (G-CIMP), confers good prognosis in these tumors [7]. Molecular subtypes of GBM have also been defined by clustering according to cell type-specific mRNA expression patterns [8,9]. Verhaak *et al.* identified classical, proneural, neural, and mesenchymal subtypes of GBM using mRNA expression, somatic mutation, and copy number data obtained from the cancer genome atlas (TCGA, [10]) [8,11]. Interestingly, clustering analysis of signature gene expression patterns of the four subtypes with expression patterns from murine neural cells showed that they are reminiscent of specific neural cell types, for example the proneural class has an oligodendrocyte rather than astrocyte signature. The proneural GBM subtype is also particularly refractory to the current standard treatment of radiotherapy and temozolomide and a very recent study by Ozawa *et al.* indicates that most GBM subtypes can arise from a common proneural-like precursor cell [12]. A consistent body of literature supports the notion that the presence of less differentiated cells in cancer confers a poorer prognosis and it may therefore be possible to identify common signatures of aggressive clinical behavior in glioma based on progenitor cell types [12-16].

In this context, microRNAs may be relevant, as changes in microRNA expression are emerging as a common feature of both neural development and glioma biology [17]. MicroRNAs are short non-coding RNAs that typically bind to the 3′ untranslated region of mRNAs and act to induce mRNA degradation or reduce translation. MicroRNAs have roles in the maintenance of brain functions throughout life and are extensively dysregulated in cancer [18,19]. In brain tumors they have been shown to promote ‘stemness’ or inhibit differentiation, consequently maintaining tumorigenesis [20]. Their expression is also altered in stem-like compartments of both brain tumors and other tumors [21-26]. In addition, microRNAs modulate neural differentiation and their expression patterns have been shown to be distinct at different cellular stages of differentiation, including oligodendrocyte precursor (OP) differentiation [27]. The presence of stem-like cells in brain cancer has been shown to be associated with more aggressive, treatment resistant tumors [13,14,16]. It is established that microRNAs have a role in maintaining a specific differentiation phenotype but it remains unclear whether prognostic microRNA signatures are exclusively tumor grade and/or molecular subtype-specific, or whether common signatures, for example associated with differentiation status, can be identified [23]. Here we have used a computational approach to test the hypothesis that differential microRNA expression profiles in groups of glioma patients with good and poor prognosis reflect changes in progenitor development pathways. We therefore correlated the microRNA expression changes between good and poor prognosis groups with microRNA expression changes in the OP differentiation pathway. Notably, OP differentiation can be modeled *in vitro* using embryonic stem cells (ESCs) that adopt an oligodendrocyte cell fate in a step-wise fashion using instructive cell culture conditions [27]. The differentiation steps include embryoid bodies (EBs), a neural progenitor cell state (NP), the oligodendrocyte progenitor stages OP1, OP2, and OP3 and the fully differentiated oligodendrocyte lineage (OL). Analysis of microRNA profiles of these cell types showed that expression changes during OP differentiation correlate with prognostic microRNA expression changes in malignant glioma. This correlation is most apparent for the OP1 cell stage, which consistently predicts survival (in >500 gliomas), hence suggesting a prognostic signature of aggressive clinical behavior that is independent of grade and malignant brain tumor subtype.

## RESULTS

### Identification of a high-grade glioma microRNA signature associated with poor patient survival

**Figure 1:**
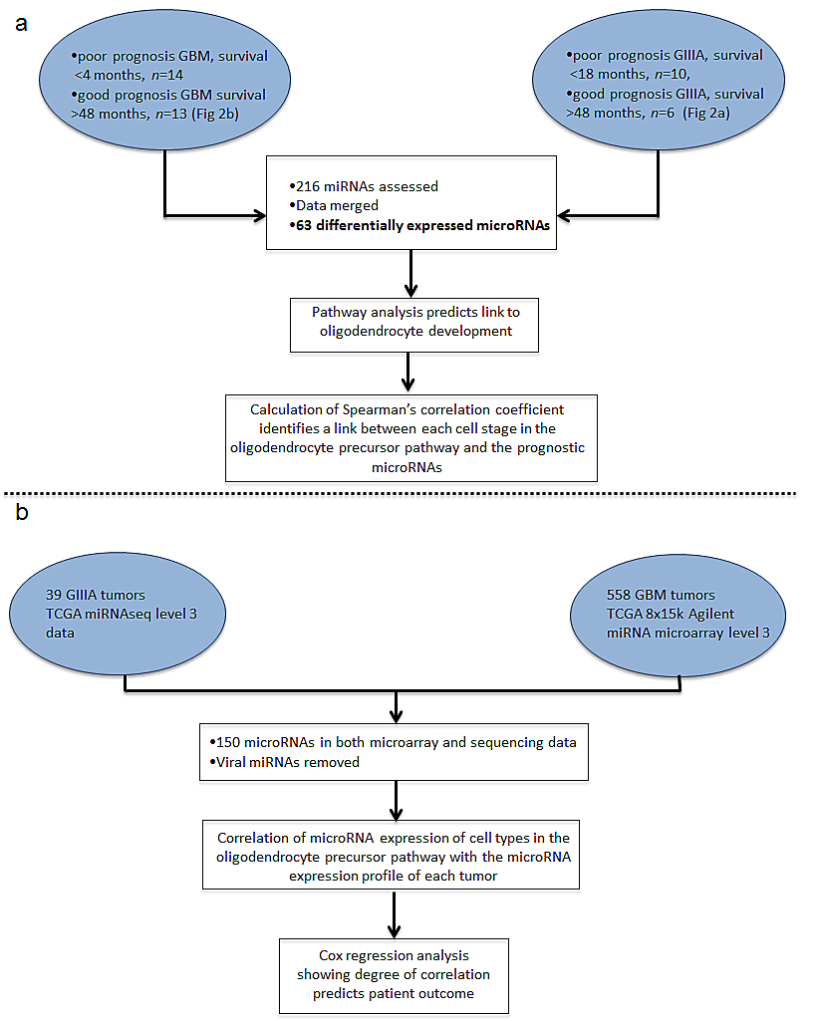
A computational analysis identifies common prognostic molecular signatures in high-grade astrocytoma (A) Differentially expressed microRNAs were identified separately in GIIIA and GBM and data were merged to create a common high-grade microRNA profile associated with prognosis. Targets of significant microRNAs were predicted and pathway analysis suggests that gene expression pathways associated with OP cells may predict patient outcome. Fold change data for each microRNA differentially expressed between each cell in the OP differentiation pathway was correlated with microRNA fold change data calculated between poor and good prognosis groups (and IDH1 mutation/IDHwt tumors) in GIIIA and GBM. (B) MicroRNA expression profiles for all 597 TCGA malignant glioma (GIIIA and GBM) were correlated with the expression values of each cell type in the OP differentiation pathway [27].

**Figure 2:**
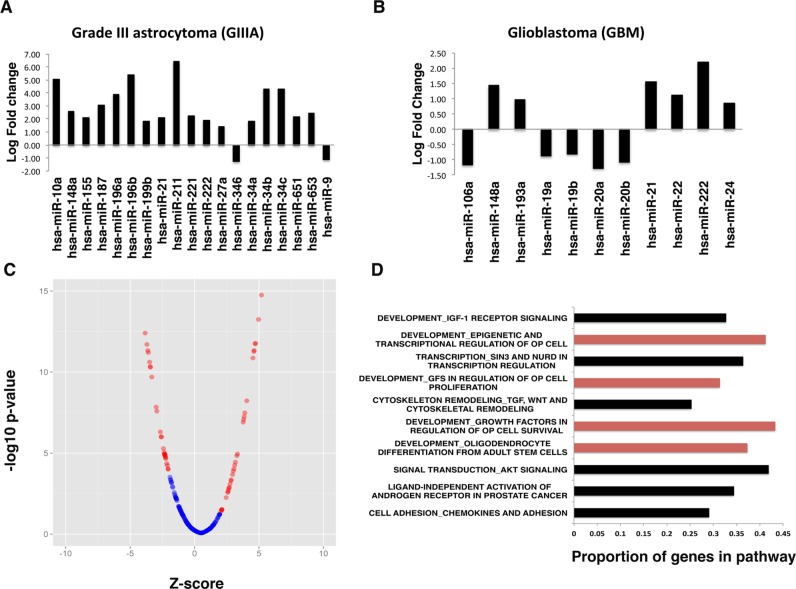
(A-B) Fold changes of the differentially expressed microRNA expression between the good and poor prognosis groups in GIIIA and GBM. (C) Plot of the microRNAs differentially expressed between good and poor prognosis groups when data from GBM and GIIIA are combined. 63 microRNAs (in red) are significantly altered between good and poor prognosis groups (p<0.05) and have a z-value of at least 2/−2. (D) The targets of the 63 microRNAs associated with patient outcome were predicted and pathway analysis revealed a significant enrichment of genes involved in several OP-related pathways.

To investigate candidate prognostic microRNAs that are associated with high-grade brain tumors (GIIIA and GBM) through a differential TCGA microRNA expression analysis, we developed the computational pipeline shown in Figure 1. Based on TCGA patient survival data [28], we defined suitable filter criteria indicative of good prognosis (>48 months for GIIIA and GBM) and poor prognosis (<10 months for GIIIA and <4 months for GBM). These cut-offs were decided by assessing the the top and bottom 10% of survival times in the TCGA cohort and including all patients with sufficient clinical and microRNA data. This yielded a total of 534 mature microRNAs from 27 GBM and 16 GIIIA tumors, respectively (Fig. 1, Table 1). Based on this dataset, we first determined the microRNAs that are differentially expressed between the good and poor prognosis groups within GBM and GIIIA specimens, separately. To minimize the false discovery rate, we used EdgeR and Limma including multiple testing correction procedures for microarray and next generation sequencing analysis [29-31]. Our approach identified 11 microRNAs that are significantly differentially expressed (with log fold changes between −1.27 and 6.39) in good versus poor prognosis groups in GBM, and 19 in GIIIAs (with log fold changes between −1.28 and 2.20). Five of the 11 candidate GBM microRNAs were lower in the poor prognosis group (Fig. 2a), whereas only 2 of the 19 GIIIA microRNAs were lower in the poor prognosis GIIIA group (Fig. 2b). The most strongly (>5 fold) increased microRNAs (miR-10a, miR-196b, miR-211) were all within the poor prognosis GIIIA group. This is consistent with previous data suggesting that miR-10a and miR-211 are implicated in progression and treatment resistance in malignant glioma [32,33].

**Table 1:** Characteristics of the grade III astrocytoma and glioblastoma TCGA tumors in poor and good prognosis groups

		GIIIA		GBM	
		Good prognosis (>48 months, *n*=6)	Poor prognosis (<18 months, *n*=10)	Good prognosis (>48 months, *n*=13)	Poor prognosis (<4 months, *n*=14)
Age at Diagnosis		40.5	59.5	41.5	61.9
Overall Survival		87.0%	8.6%	83.4%	2.7
Gender	Male	67%	40%	62%	50%
	Female	33%	60%	38%	50%
IDH1 mutation status	Mutated	100%	10%	0%	0%
	WT	0%	90%	100%	100%

Overall, our intra-grade glioma microRNA comparison of good and poor prognosis only yielded three microRNAs, the oncomiR miR-21, the apoptosis regulator miR-148a, and the tumor suppressor regulator miR-222 that could serve as candidate predictors of poor prognosis in both GBM and GIIIA [34-36]. This low overlap between GBM and GIIIA candidate prognostic microRNAs raises the question as to whether it is possible to identify a common microRNA signature for high-grade glioma, or whether the statistical power of the intra-grade comparison approach is insufficient to reveal a GBM/GIIIA poor prognosis signature. To address this question and to increase statistical power in our differential microRNA expression analysis, we combined the z-values (Z_r,combined_) from the good and poor prognosis groups of GIIIA (Z_r,III_) and GBM (Z_r,IV_) accounting for differences in microRNA expression profiling platforms using a suitable computational algorithm based on the formula for each microRNA, r, including fold change, FC, and standard error (SE):

$${\text{Z}}_{\text{r},\text{III}}=\log({\text{FC}}_{\text{r},\text{III}})/{\text{SE}}_{\text{r},\text{III}}{\text{Z}}_{\text{r},\text{IV}}=\log({\text{FC}}_{\text{r},\text{IV}})/{\text{SE}}_{\text{r},\text{IV}}$$

$${\text{Z}}_{\text{r},\text{combined}}=({\text{Z}}_{\text{r},\text{III}}{\text{+Z}}_{\text{r},\text{III}})/\sqrt{2}$$

Under the null hypothesis that Z_r,III_ and Z_r,IV_ are both N(0,1) and independent, Z_r,combined_ will also be N(0,1) and can therefore be interpreted as a Z value.

This approach yielded a pool of 216 microRNAs whose differential expression was analyzed across all relevant poor/good prognosis GBM and GIIIA TCGA specimens, thereby creating z-scores and p-values for the individual microRNAs. Using the multiple testing corrected p-values for each microRNA yielded 63 microRNAs that significantly change expression between good and poor prognosis high-grade gliomas as indicated by a >2 fold change of standard deviations from the mean microRNA fold change (FDR<0.05) (Fig. 2c).

Our results are consistent with a hypothesis that a pool of 63 microRNAs form part of a molecular network that is associated with and/or drives aggressive clinical behavior in high-grade gliomas. To identify the molecular pathways that are likely regulated by the 63 candidate prognostic microRNAs, we predicted their mRNA targets using standard bioinformatic approaches. In order to focus on the mRNA targets that are involved in prognosis, we first enriched for those that are associated with either good or poor prognosis. We compared good prognosis and poor prognosis mRNAs in GIIIA and GBM (Table 1) using the same criteria as those described above for microRNA analysis. The mRNA data (z-scores and p-values) for GIIIA and GBM were merged resulting in 4259 mRNAs with significant (p<0.05) >2 fold changes. The targets of the 63 microRNAs associated with patient outcome were predicted from the 4259 mRNAs using the target prediction databases Miranda, Pictar and Targetscan [37-39]. We only used targets that were present in at least two of these databases, resulting in 1618 predicted targets for the microRNAs (Supplementary list S1). Subsequently, we entered these mRNAs into the Metacore software and carried out a pathway analysis revealing significant enrichment of genes involved in several cancer-related pathways (Fig. 2d). These pathways included IGF and AKT signalling, epigenetic and transcriptional regulation, growth factor, androgen and chemokine-effectors, and cytoskeletal remodeling. Interestingly, four of these pathways are linked with OP cell fate decisions such as survival, proliferation, differentiation, and myelination. This provides correlative evidence to suggest that the microRNAs associated with survival in high-grade glioma have roles in OP differentiation pathways.

### OP gene expression signatures correlate with poor prognosis in glioma

**Figure 3:**
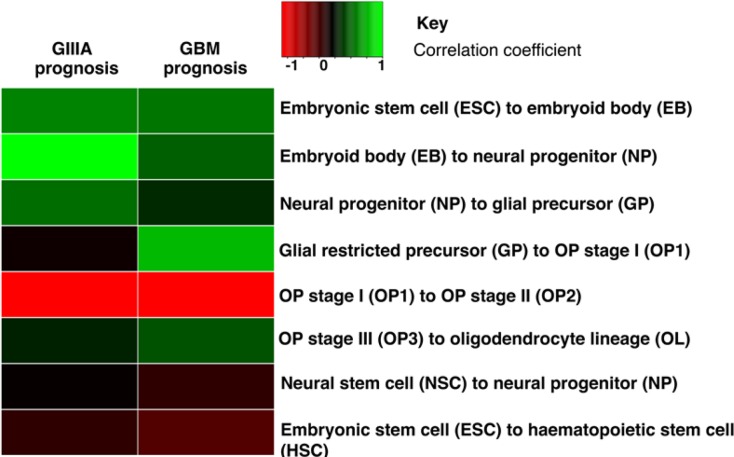
Correlation coefficients comparing the fold change of microRNA expression between each stage in the OP pathway and the GIIIA and GBM good and poor prognosis groups The top 6 rows relate to data from Letzen *et al.* and the bottom rows refer to data from Goff *et al.* and Risueño *et al*. [27,40,41]. The highest negative correlation is the transition from OP1 to OP2 and the highest positive correlation is the transition from GP to OP1.

To further determine whether the activity of microRNAs in different cell stages of the OP differentiation pathway are associated with malignant glioma patient outcome, we accessed published data describing microRNA profiles associated with stages in the differentiation of ESCs into oligodendrocytes [27]. Our initial hypothesis was that presence of less differentiated oligodendrocyte cells in glioma confers a poorer prognosis. To this end, we questioned whether microRNA expression changes throughout OP differentiation resemble the prognostic microRNA expression pattern of malignant glioma. First, we calculated fold changes between each progenitor cell type in the OP differentiation pathway and correlated these with the fold differences between poor prognosis and good prognosis samples of GIIIA or GBM (Fig. 2a-b). Only microRNAs that are significantly differentially expressed between each stage of the OP differentiation pathway and with at least a 2-fold change in expression were used. The OP2 to OP3 stage was omitted, as there were too few differentially expressed microRNAs between these cell types. In GIIIA, the microRNA expression differences between good and poor prognostic cases correlated directly with the changes associated with differentiation from NP to GP (correlation coefficient = 0.50, p<0.05), which was not evident in GBMs. In both grades, the expression differences between good and poor prognosis showed a negative correlation with the changes associated with differentiation from OP1 to OP2 (correlation coefficient −0.54 for GIIIA and −0.47 for GBM, p<0.05) (Fig. 3). Next, we tested whether these correlations are a result of non-specific correlations with any ESC differentiation pathway (including non-neural lineages), or whether these high correlations are specific for neural differentiation. We used expression data from a study comparing microRNA expression between ESC cells and hematopoietic progenitors (HPs) and between neural stem cells (NSCs) and neural progenitors (NPs) [40-41]. We correlated the differences in the differentially expressed microRNAs between ESCs and HPs and NSC and NP cells with the differential microRNA expression patterns between good and poor prognosis in GIIIA and GBM. This approach revealed no significant correlations (p>0.05, ρ correlation coefficient <0.15) indicating that the microRNA expression differences between good and poor prognosis of malignant glioma are specifically correlated with microRNA expression changes in OP differentiation, and not with other differentiation pathways (Fig. 3).

A notable difference in good and poor prognosis GIIIA patients studied here was their IDH mutation status (Table 1), which is used to classify patients clinically; those with the mutation are usually proneural tumors and have a favorable prognosis [5,6]. In our cohort, all the good prognosis patients had an IDH mutation, while only one poor prognosis patient was IDH mutated. It could be argued that the difference in microRNA expression between these two groups is simply due to different biology associated with the presence or absence of an IDH mutation. To test this possibility, we obtained sequencing data for IDH mutated (IDHmut, *n*=139) and IDH wild-type (IDHwt, *n*=39) gliomas (combining data for both grade II and III glioma for added statistical power) from the TCGA and determined microRNA expression differences between the two groups using the criteria previously stated. The microRNA expression fold differences between IDHwt and IDHmut were correlated with the fold changes between each stage in the OP differentiation pathway. The only significant correlation observed was an inverse correlation between IDHmut and IDHwt and OP1 to OP2. Good versus poor prognosis GIIIA and GBM also correlated with this OP differentiation stage. Critically, the fold differences between IDHmut and IDHwt did not correlate with the changes during differentiation from NP to GP (p<0.05, ρ correlation coefficient < −0.34). Therefore we conclude that the correlation we have shown between prognosis and OP stage differentiation is independent of IDH mutation status.

### Correlation of microRNA expression with the OP cell stage is associated with glioma survival

Correlations of the microRNA expression differences between good and poor prognosis cases and between neural differentiation stages imply that correlation with the OP1 cell type is most closely related to prognosis.

**Figure 4:**
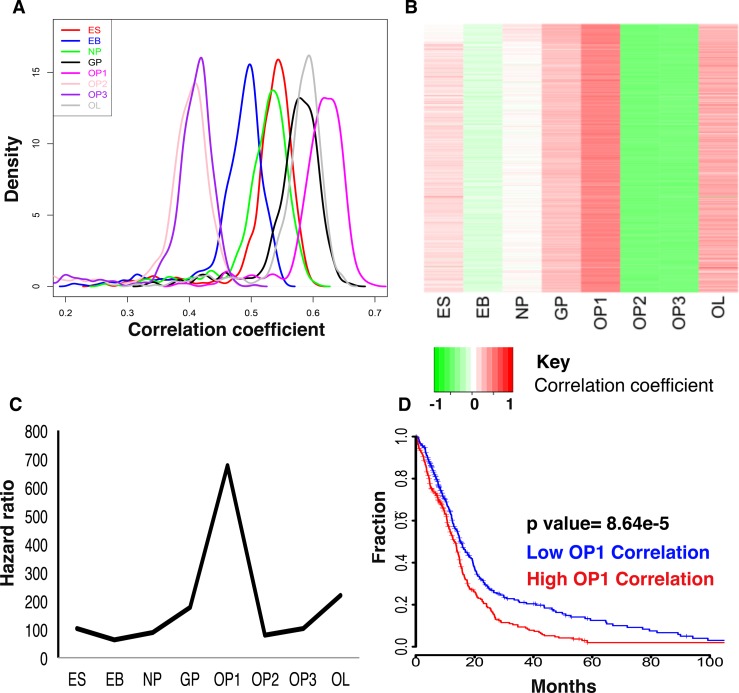
(A) Density plots of the Spearman's correlation coefficients for each cell type with all GIIIA and GBM tumors in the TCGA. All cell types in the OP differentiation pathway show positive correlation of microRNA expression with each tumor. The oligodendrocyte lineage and OP1 cells show the most significant positive correlations with the tumors. (B) Heatmap of correlation of each GIIIA/GBM tumor with each OP cell type. (C) Hazard ratios from Cox regression analysis of the correlation patterns of each cell type shows that OP1 microRNA expression correlation is the most predictive in terms of prognosis. MicroRNA profiles of all cell types were significantly associated with survival (p<0.05); however, there is a peak in statistical power when OP1 cells are used as the predictor. (D) Kaplan Meier plot of the OP1 correlation coefficients for grade III and IV gliomas. Groups are separated above and below the median correlation of microRNA expression between OP1 and tumor.

In order to examine this hypothesis, we correlated the microRNA expression profiles of each differentiation stage in the oligodendrocyte differentiation pathway with microRNA expression profiles of 39 GIIIA, 558 GBM and 10 non-tumor samples from the TCGA. Only microRNAs present in all platforms (sequencing and microarray) were used (150 microRNAs) (Supplementary list S2). The majority of the 597 tumors positively correlated with each cell type in the OP differentiation pathway. Seven GIIIA tumors did not correlate with OP2 or OP3 microRNA expression, and two GIIIA tumors did not correlate with OP2 expression. Across all tumors assessed, the highest correlations were with OP1 and oligodendrocyte lineage (OL) cell types microRNA expression patterns (Fig. 4a and b). The cell type that correlated most positively with tumors was OP1. This cell type was most correlated with each tumor type and also ten non-tumor samples.

Twenty-three GBMs had the highest correlation with OL and one GBM had the highest correlation with glial restricted precursor (GP). Average correlation with OP1 was 0.60 for GIIIA, 0.93 for GBM, 0.69 for the mesenchymal subtype of GBM (n=155), 0.67 for the classical the GBM subtype (n=143), 0.36 for G-CIMP GBM subtype (n=38), 0.54 for neural GBM (n=82), 0.58 for proneural GBM (n= 97) [1,6,11], and 0.88 for non-tumor samples [11]. These results indicate that GBM is most positively correlated with OP expression patterns.

To determine the association of the OP differentiation cell stages with high-grade glioma patient survival, the correlation values for each of the eight cell types in the OP differentiation pathway with all 597 tumors in the TCGA were assessed for association with survival using Cox regression analysis. Rho (ρ) values (Spearman's coefficient) for all cell types were significant negative predictors (p<0.05) of survival. The highest hazard ratio was for correlations with the OP1 cell type (Fig. 4c), which indicates that gliomas with microRNA expression patterns similar to OP1 cells have a poorer patient outcome (Cox regression HR = 13.02, 95% CI = 3.77-45.04, p = 5.02e-05) (Fig. 4d). Taken together, our results suggest that the most aggressive malignant gliomas (both GIIIA and GBM) have a microRNA expression pattern that aligns with expression patterns characteristic of the OP1 cell stage.

## DISCUSSION

### Prognostic glioma microRNAs align with OP pathways

There has been considerable discussion over subtyping of GBM based on expression and copy number data. However, so far this approach has not delivered robust clinical biomarkers and the field is further complicated by data confirming that subtypes can co-exist within the same tumor thereby creating a diversity of oncogenic transcriptional programs that contribute to treatment resistance [42,43].

Models of glioma suggest these tumors may be defined by the initiating cell type or the type of initiating mutation [44]. Despite these observations, the glial cell of origin in different histological types of glioma remains unclear [45]. It has been proposed that OPs may fill this role in some subtypes and this is supported by data suggesting that mesenchymal GBM can arise from a proneural-like precursor [12, 46].

Using integrated mRNA and microRNA expression data we have identified that prognostic microRNA expression patterns in malignant glioma correlate with microRNA expression changes during oligodendrocyte differentiation (Fig. 2d). Our study is novel in identifying grade-independent and subtype-independent prognosis prediction using microRNAs as biomarkers, which are stable in clinical samples, with additional validation, and may be appropriate for implementation into clinical practice [47].

MicroRNA expression changes associated with cellular transitions between OP1 and OP2 (GIIIA and GBM, Fig. 3) suggest that more aggressive tumors have more cells with OP1-like expression patterns, or are simply less differentiated.. Whether these are non-malignant OP1s present within the tumor, or simply less differentiated cells, mass, malignant cells with similarities to these cells cannot be ascertained from our data. The tumor samples under study here were defined by the TCGA as having at least 70% tumor nuclei which suggests this is unlikely to be a non-malignant population of cells.

In line with of our computational results, OPs have been shown to stimulate a more aggressive phenotype by promoting neo-vascularization of glioma and are present at the invasive front of high grade tumors [48]. Initial neoplasia-generating aberrations in NSCs can only become transforming upon differentiation into an OP, suggesting that these cell types are important in tumor initiation, as well as defining its behavior [45]. Supporting this notion, both proneural and mesenchymal tumors have been shown to arise from a common precursor [12,45]. OP cells are also implicated in maintaining self-renewal by means of asymmetric cell division, supporting both self-renewal and proliferation in the tumor [49]. These cells are also defined by their PDGFRA expression, and recent studies show that amplification of this is an initiating event in gliomagenesis [12,50,51].

### Translational relevance of the OP1 prognostic signature

Our results suggest a more OP1-like phenotype is associated with a more aggressive tumor (Fig. 4c-d), and presence of these OP1 microRNA patterns predicts poorer prognosis. MicroRNA signatures predicting more aggressive tumors have been developed in the past, yet their relevance to tumor biology is not well understood [52-54]. Also, subtype-specific signatures are not easy to implement into the clinical routine of standard healthcare laboratories due to logistic challenges (i.e. multiple testing procedures) and the need for diverse state-of-the-art profiling platforms (i.e. next generation sequencing) as well as high-level bioinformatics/computational support. Hence, it would be desirable to replace complex prognostic signatures with a few key biomarkers wherever possible. For example, Letzen *et al* describe peaks of miR-10a and miR-21 expression in OP cells [27] that were both significantly increased in our merged data compared to other neural cell types and it may therefore be possible that these microRNAs alone have the potential to be exploited as biomarkers for the presence of OP-like cells. Prospective observational clinical trials will be needed to address this hypothesis.

Taken together, we provide preliminary data that classification of malignant glioma based on microRNA expression patterns seen in OPs may predict the outcome of the disease, which could not only inform patient management but also guide development of novel treatments. The statistical power of future work is likely to be increased due to the availability of more samples in TCGA and other repositories. This is also a principle that could be extended to other tumor types, to elucidate the characteristic microRNA profiles exhibited in particular by poor prognosis tumors.

## MATERIALS AND METHODS

### MicroRNA and mRNA expression analysis

All computational work was performed in R (v2.15.1). Level 3 Agilent microRNA 8×15k microarray, G4520A microarray gene expression data and clinical information for GBM and non-tumor samples were downloaded from The Cancer Genome Atlas (TCGA) [28]. Level 3 Illumina HiSeq sequencing data for mature microRNA and mRNA expression plus clinical information for lower grade gliomas were also downloaded from TCGA. Due to the differences in normalization methods and quantification artifacts of microarray and sequencing platforms, the expression changes within the grades were ascertained using appropriate statistical packages developed for microarrays and sequencing [29,30]. Then these data were merged, rather than direct merging of the data prior to differential expression analysis. The good and poor prognosis groups of these glioma datasets were selected according to the published survival data in the TCGA database (Table 1). EdgeR was used to compare microRNA and mRNA expression between the two GIIIA survival groups and 139 IDH mutated and 39 IDH wild-type grade II and III tumors [29]. The linear Models for Microarray Data (LIMMA) package was used to compare microRNA expression for the poor and good prognosis groups in GBM [30].

For each microRNA or mRNA, r, the z-scores associated with GIIIA (III) and GBM (IV) prognosis were calculated separately from their log(fold change, FC) and corresponding standard error, SE:

$${\text{Z}}_{\text{r},\text{III}}=\log({\text{FC}}_{\text{r},\text{III}})/{\text{SE}}_{\text{r},\text{III}}{\text{;Z}}_{\text{r},\text{IV}}=\log({\text{FC}}_{\text{r},\text{IV}})/{\text{SE}}_{\text{r},\text{IV}}$$

Under the joint null hypothesis, log(FC_r,III_) = log(FC_r,IV_) = 0, the two z-scores are N(0,1) distributed and independent, so the sum Z_r,III_ + Z_r,II_ is N(0,2). The p-values corresponding to the joint null hypothesis were adjusted for multiple testing using the Benjamini-Hochberg method [31].

### Pathway prediction

Miranda, Pictar and Targetscan were used to predict targets for differentially expressed microRNAs from the differentially expressed mRNAs using the RmiR package [37-39, 55]. Targets were only considered if they were present in at least two of these databases. The resulting targets were entered into the pathway analysis program Metacore® (Thomson Reuters).

### Analysis of the differentiation pathway

We used data published in Letzen *et al,* which describes the microRNA expression fold changes between each cell differentiation stage within the OP differentiation pathway including embryonic stem cells (ESCs), neural embryoid bodies (EB), neural progenitors (NP), glial restricted precursors (GP), oligodendrocyte precursors (OP) I, OP II, OP III and the oligodendrocyte lineage (OL) [27]. Spearman's correlation was performed on the fold change between good and poor prognosis groups within GIIIA and GBM, with the expression changes of the significantly differentially expressed microRNAs with at least 2-fold change at each stage in the OP differentiation pathway. The fold changes of all microRNAs of significance between OP cell types were used, regardless of their significance for survival. As a control, expression values from Taqman PCR microRNA expression between ESCs and hematological precursors (HP) as described in Risueño *et al.* and between neural stem cells (NSCs) and NPs as described in Goff *et al.* were used to calculate the ß Ct and perform Spearman's correlation with the prognosis-associated fold differences in GIIIA and GBM [40-41]. Only microRNAs significantly differentially expressed between the ESCs and HPs were used (139 microRNAs) in the correlation analysis.

### Correlation of microRNA expression of the OP pathway with malignant glioma tumors

Microarray expression data was processed as described in Letzen *et al.* [27] using Agilent Feature Extraction software and the gTotalGeneSignal was correlated with the level 3 expression data from the TCGA GIIIA astrocytoma (*n* =39), GBM tumors (*n*=558) and non-tumor samples (*n*=10). Only microRNAs detected on all platforms (Agilent microarray G4470C and custom TCGA Agilent microarray, and Illumina HiSeq sequencing) were included resulting in 150 microRNAs. GBMs were classified according to Brennan *et al* [11]. The correlation patterns of each cell type for every tumor was analyzed for association with survival using Cox regression and log-rank tests.

## SUPPLEMENTARY MATERIAL


